# Association between brain-derived neurotrophic factor and symptoms of insomnia and depression in inflammatory bowel disease (IBD) patients

**DOI:** 10.1192/j.eurpsy.2021.1478

**Published:** 2021-08-13

**Authors:** M. Sochal, P. Bialasiewicz, A. Gabryelska, J. Fichna, R. Talar-Wojnarowska, E. Małecka-Panas

**Affiliations:** 1 Department Of Sleep Medicine And Metabolic Disorders, Medical University of Lodz, Poland, Lodz, Poland; 2 Department Of Biochemistry, Medical University of Lodz, Lodz, Poland; 3 Department Of Digestive Tract Diseases, Medical University of Lodz, Lodz, Poland

**Keywords:** BDNF, Insomnia, psychosomatics, inflammatory bowel disease

## Abstract

**Introduction:**

Brain-derived neurotrophic factor (BDNF) plays an important role in depression and sleep disorders. It influences the inflammatory process and may affect the interactions between psychological state and gastrointestinal symptoms.

**Objectives:**

The study aimed to compare BDNF concentrations in the group of Crohn’s disease (CD), ulcerative colitis (UC) patients, and healthy control (HC), as well as to correlate it with the severity of depression and insomnia.

**Methods:**

The study included 94 inflammatory bowel disease patients (IBD, 57 CD, and 37 UC) and 26 HC. Each participant completed the following questionnaires: Pittsburgh Sleep Quality Index (PSQI), Athens insomnia scale (AIS), and Beck Depression Inventory (BDI). BDNF protein concertation measurements were performed using ELISA. Funding: National Science Centre, Poland-2018/31/N/NZ5/03715.

**Results:**

CD patients had a higher serum level of BDNF (22.5 ng/mL, IQR:17.5-28.5) than UC patients (19.1 ng/mL, IQR:12.3-24.6; p=0.045). CD group had higher BDNF concentrations than HC (17.5 ng/mL, IQR:13.2-23.8; p=0.010), but no such differences were found between UC and HC groups (p=0.544). A positive correlation was found between AIS and BDNF among IBD (r=0.22, p=0.035). Additionally, patients, who obtained high BDI scores (>7 points) had lower BDNF concentrations than others (p=0.004). The patients with long sleep latency (>10 min) achieved a higher BDNF level than others (p=0.038). However. BDNF level did not correlate with PSQI results.

**Conclusions:**

BDNF serum level is increased in CD, but not in UC patients. Overall, the severity of insomnia symptoms correlates positively with BDNF levels. Future research should focus on the further explanation of those observations.

